# Supplementary material for: Progesterone receptors drive advanced breast cancer phenotypes including circulating tumor- and stem-like cell expansion in the context of ESR1 mutation
Source: NPJ Breast Cancer. 2026 Apr 7;12:75. doi: 10.1038/s41523-026-00939-8 (PMC13233901; doi:10.1038/s41523-026-00939-8)

SUPPLEMENTARY MATERIAL

SUPPLEMENTARY FIGURES AND LEGENDS

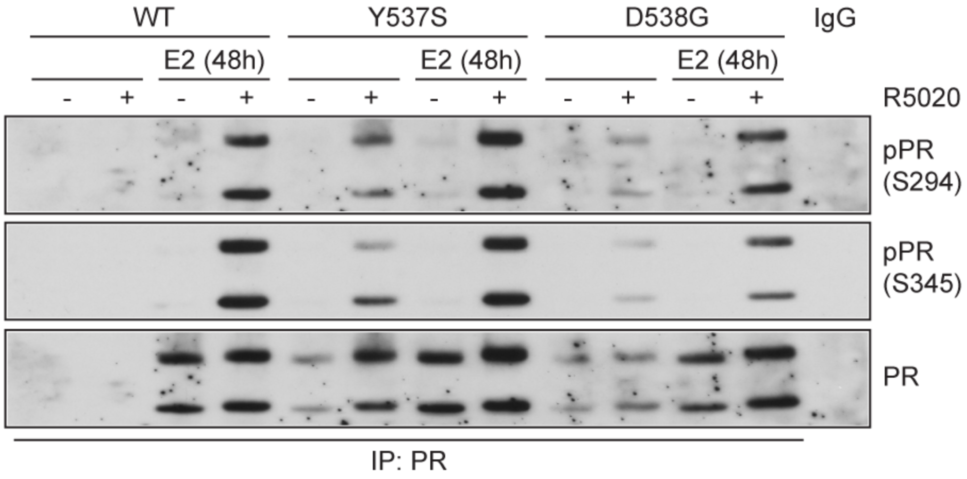

Supplementary Figure 1. PR phosphorylation levels in MCF7 ER cells. Immunoprecipitation of PR in MCF7 ER (WT, Y537S, D538G) cells pre-treated with E2 (1 nM) for 48 h followed by treatment with veh (EtOH) or R5020 (10 nM) for 60 min.

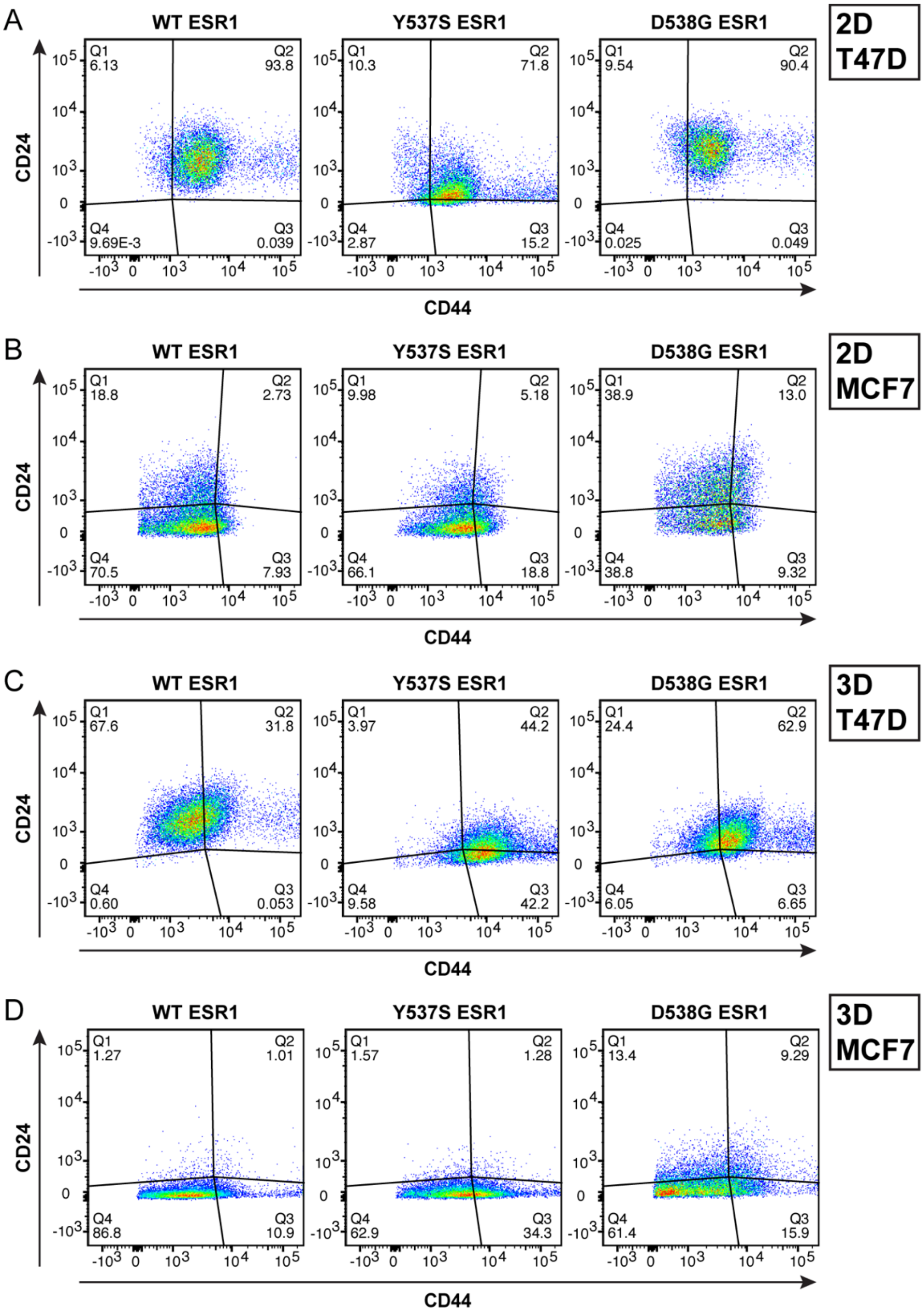

Supplementary Figure 2. Representative flow cytometry dot plots shown for CD44^{hi}/CD24^{lo} populations in T47D and MCF7 ER cells cultured in 2D (adherent) (**A, B**) and 3D (tumorsphere) (**C, D**) conditions.

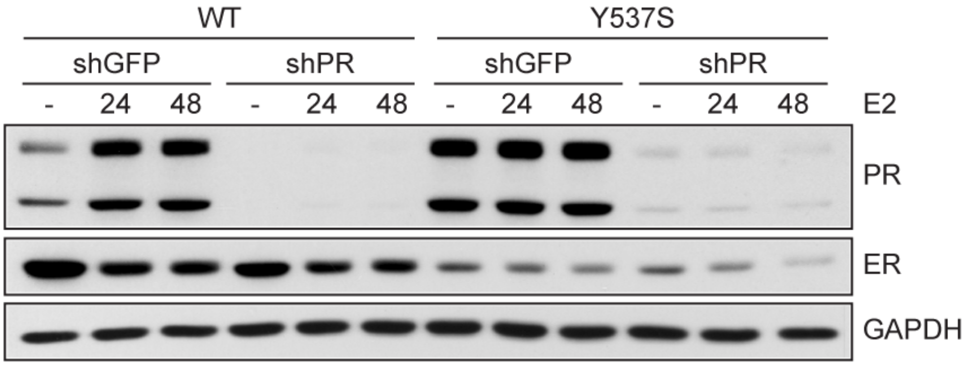

Supplementary Figure 3. Western blot of PR knockdown in T47D ER (WT, Y537S) cells treated with E2 (1 nM) for 24 and 48 h.

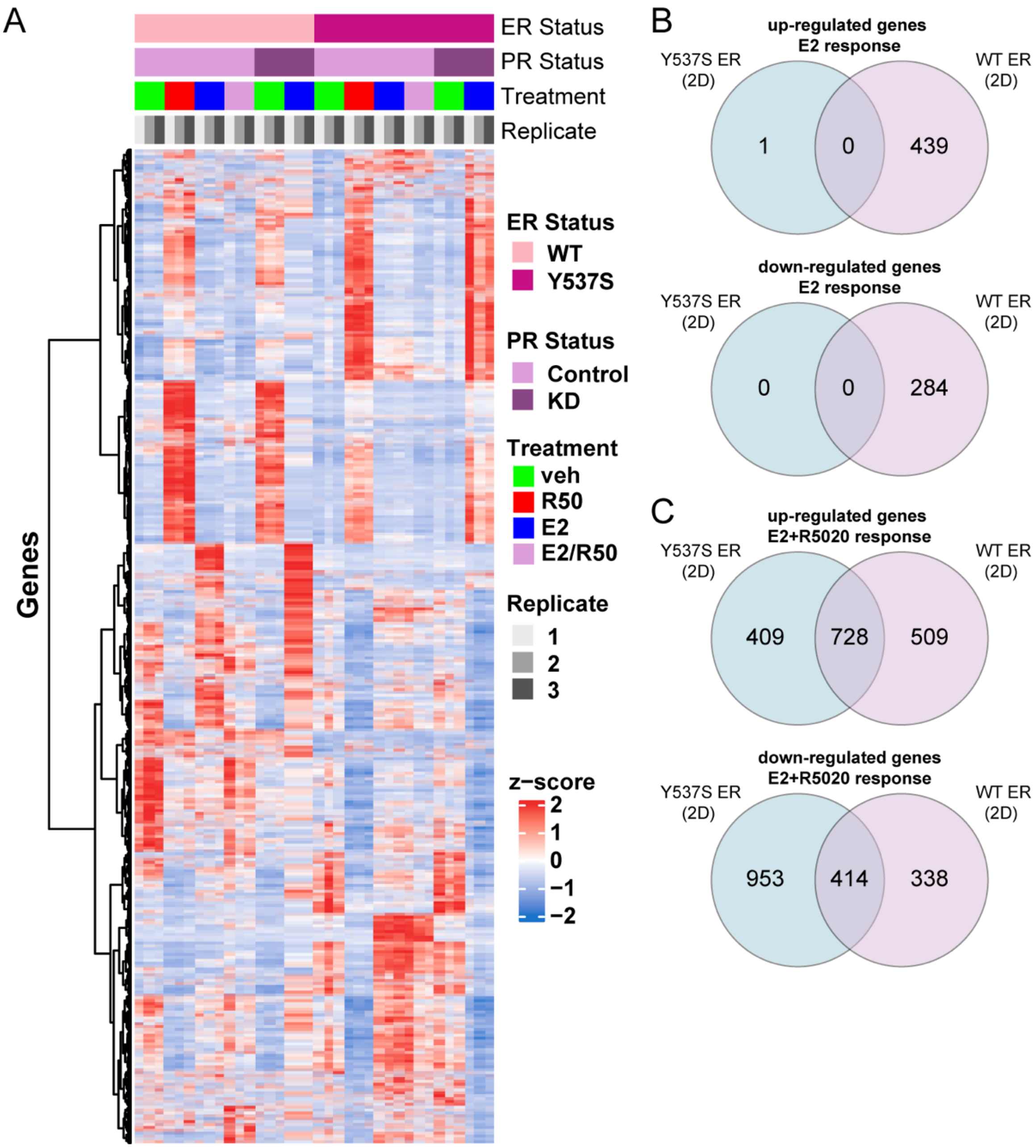

Supplementary Figure 4. (**A**) Hierarchal clustered heat map of 2D samples. Venn diagrams showing differentially expressed genes up or downregulated >4-fold in response to (**B**) E2 (1 nM) or (**C**) E2 (1 nM) plus R5020 (10 nM) combination.

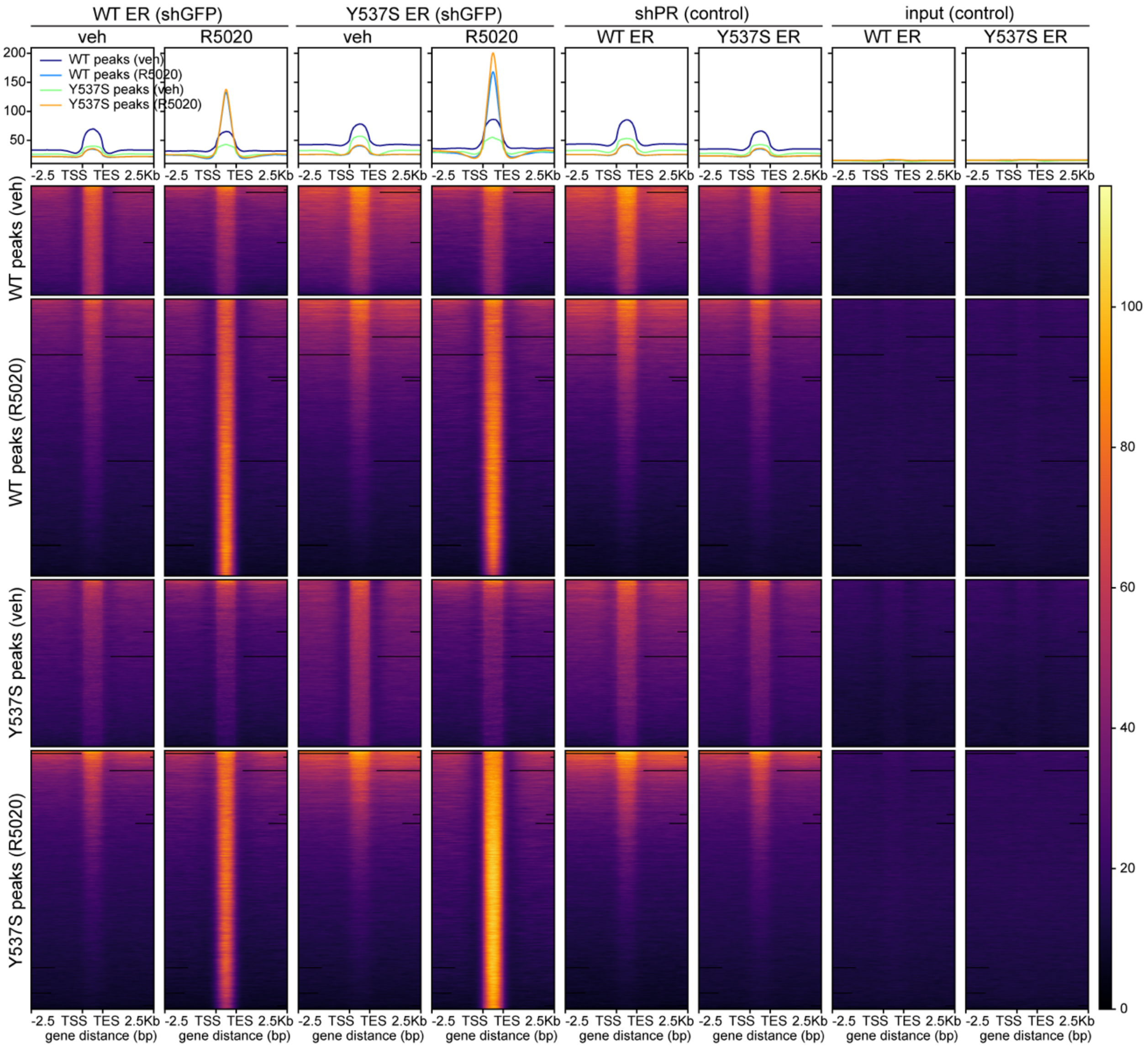

Supplementary Figure 5. Phospho-PR ChIP-seq signal in WT and Y537S ER cells in response to R5020 (10 nM). shPR and input samples are included to show enrichment compared to negative controls.

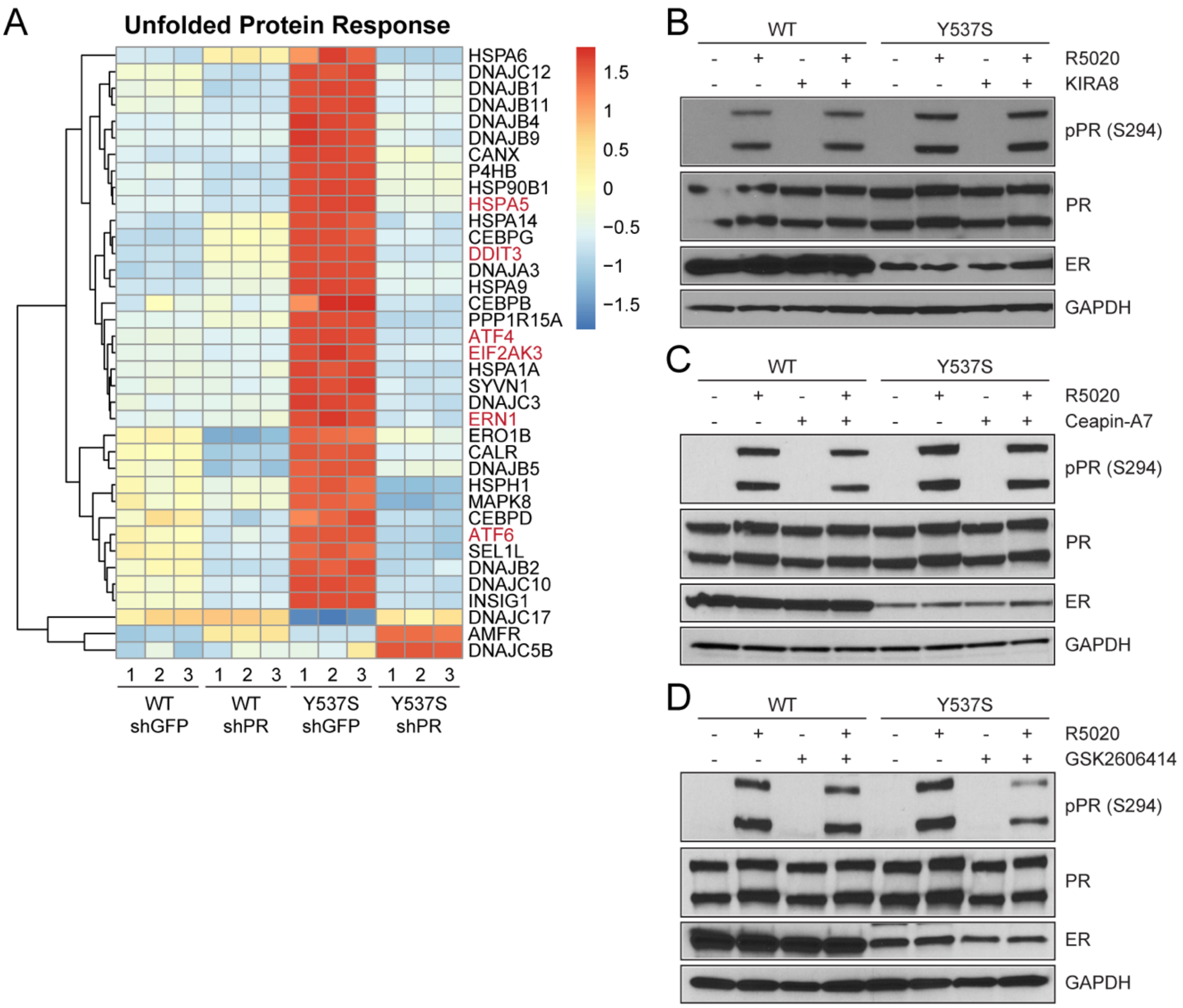

Supplementary Figure 6. (**A**) Heatmap showing select differentially expressed genes (DEGs) of the UPR pathway from Ingenuity Pathway Analysis from RNA-seq studies performed in 3D cultured T47D WT and Y537S (shGFP, shPR) cells. Key UPR pathway components are indicated in red text (*ATF6, ATF4, ERN1* [IRE1 alpha], *EIF2AK3* [PERK], *HSPA5* [BiP], *DDIT3* [CHOP]). Western blot of T47D ER cells (WT, Y537S) pre-treated with vehicle, (**B**) KIRA8 (5 µM), (**C**) Ceapin-A7 (5 µM), or (**D**) GSK2606414 (5 µM) for 2h followed by vehicle or R5020 (10 nM) for 1h.

SUPPLEMENTARY MATERIAL

UNPROCESSED ORIGINAL IMAGES OF WESTERN BLOTS (SCANNED FILMS)

Figure 1A

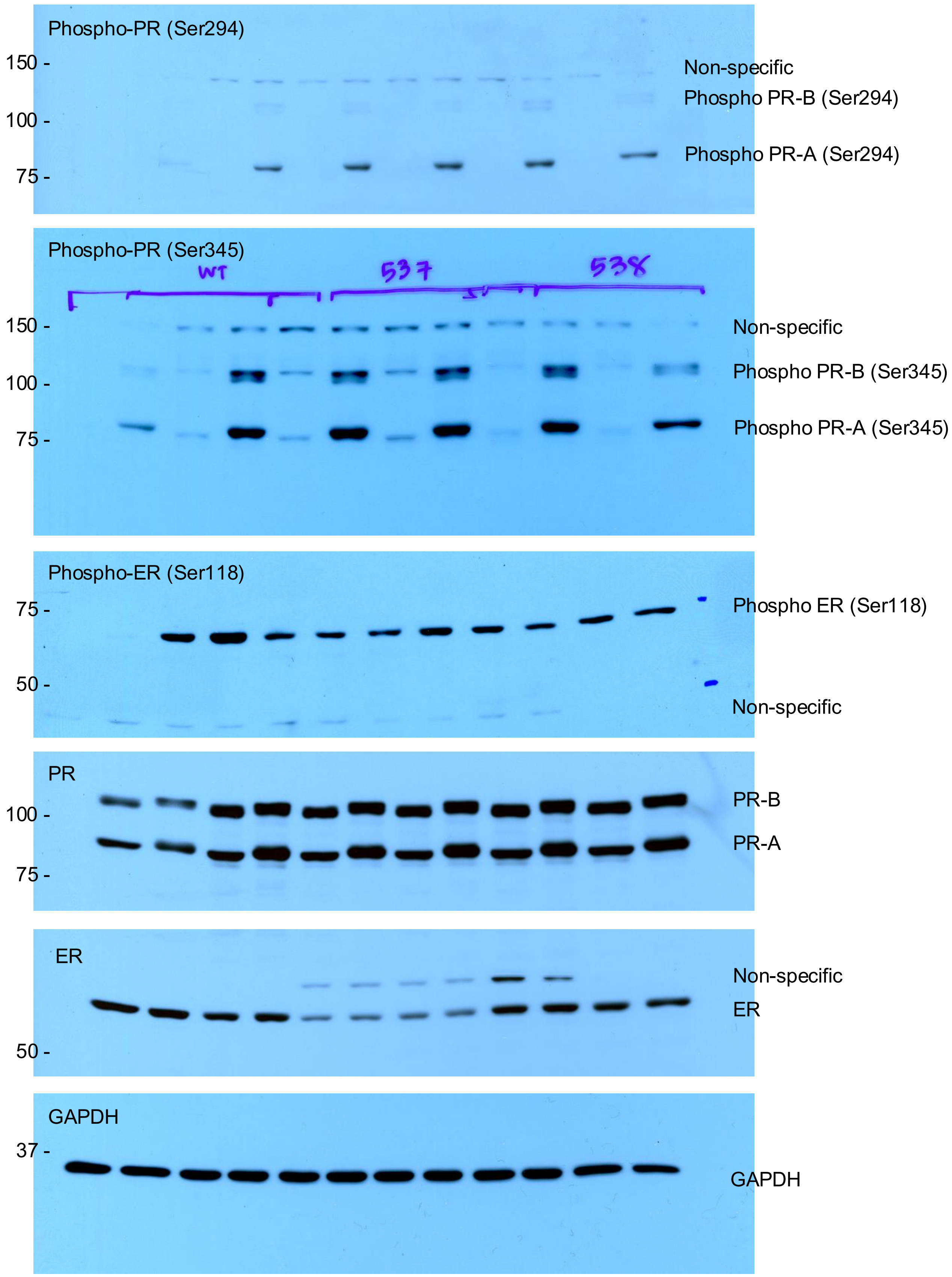

Phospho-PR (Ser294)
150 -
100 -
75 -
Non-specific
Phospho PR-B (Ser294)
Phospho PR-A (Ser294)
Phospho-PR (Ser345)
WT
537
538
150 -
100 -
75 -
Non-specific
Phospho PR-B (Ser345)
Phospho PR-A (Ser345)
Phospho-ER (Ser118)
75 -
50 -
Phospho ER (Ser118)
Non-specific
PR
100 -
75 -
PR-B
PR-A
ER
50 -
Non-specific
ER
GAPDH
37 -
GAPDH

Figure 1B (co-immunoprecipitation blot)

ER (IP: PR)

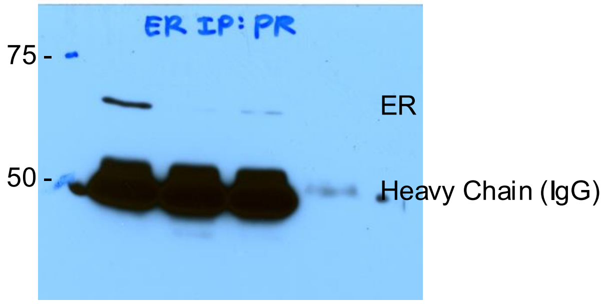

PR (IP: PR)

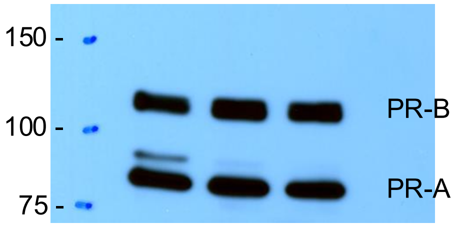

Figure 1B (whole cell lysate)

ER

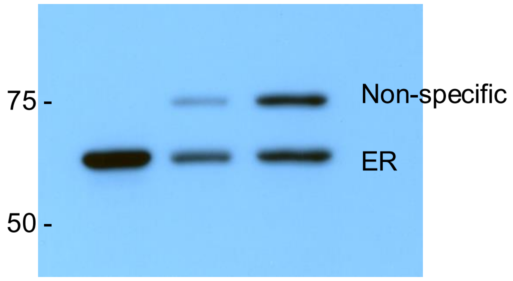

PR

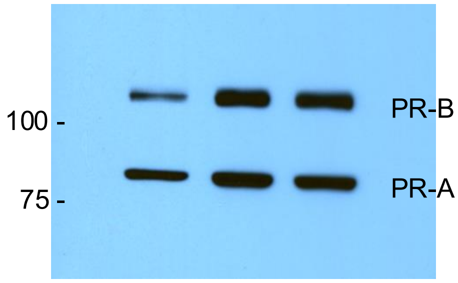

GAPDH

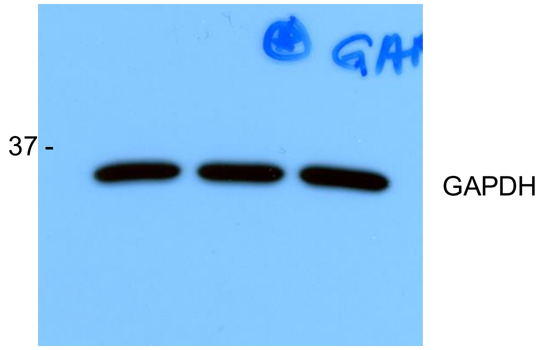

Figure 1C (co-immunoprecipitation blot)

ER (IP: PR)

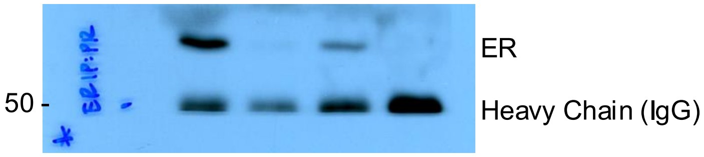

PR (IP: PR)

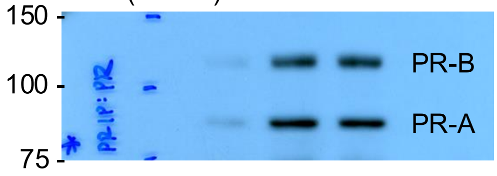

Figure 1C (whole cell lysate)

ER

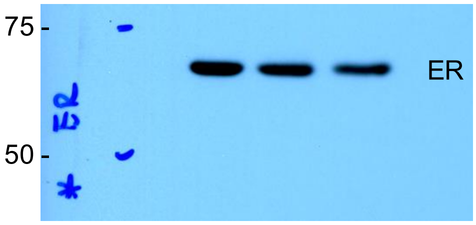

PR

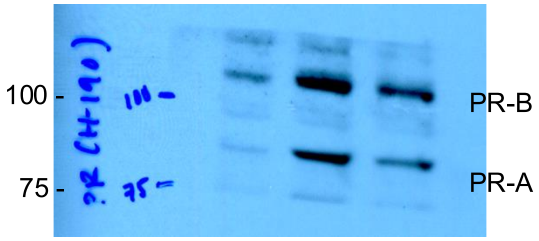

GAPDH

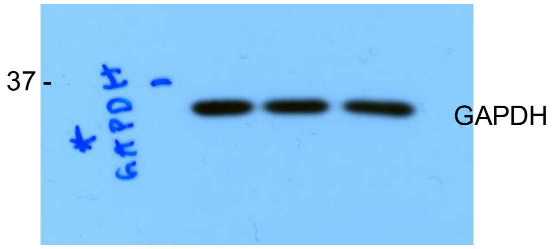

Figure 3C

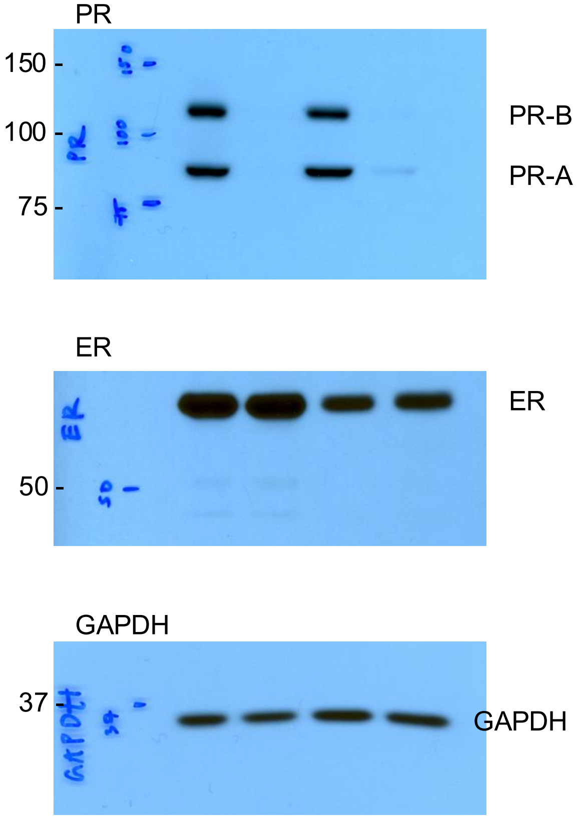

Figure 6E

Phospho PR (Ser294)

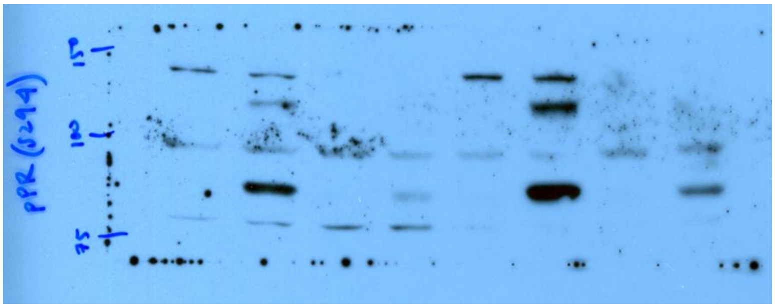

Non-specific
Phospho PR-B (Ser294)
Phospho PR-A (Ser294)

PR

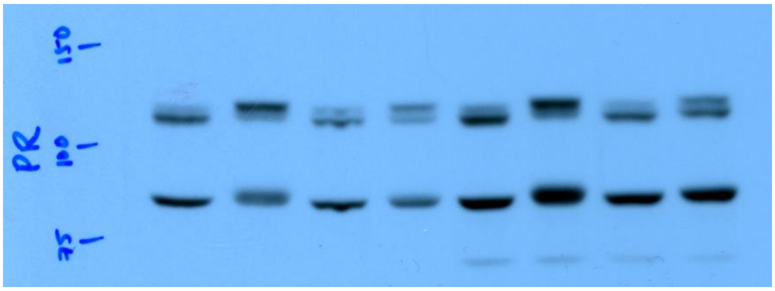

PR-B
PR-A
Non-specific

ER

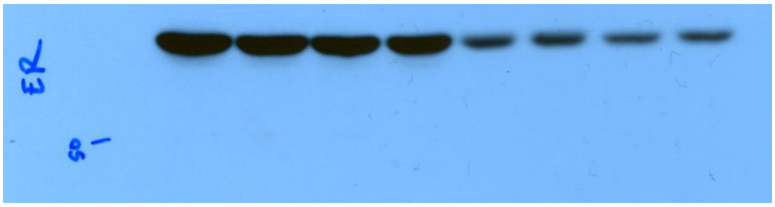

ER

GAPDH

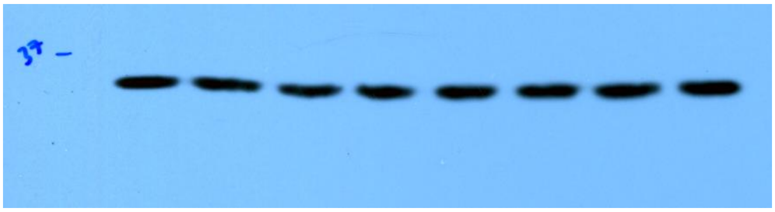

GAPDH

Supplementary Figure 1

Phospho PR (Ser294), IP: PR

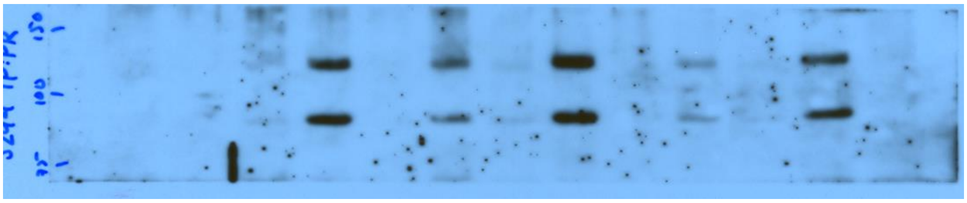

Phospho PR-B (Ser294)

Phospho PR-A (Ser294)

Phospho PR (Ser345), IP: PR

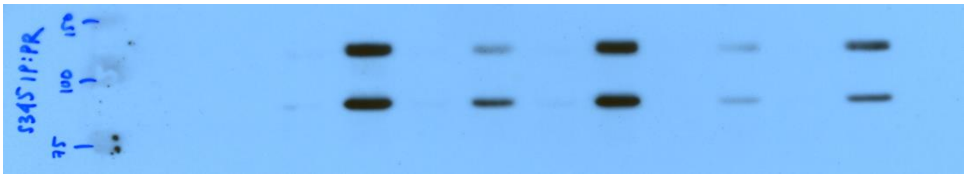

Phospho PR-B (Ser345)

Phospho PR-A (Ser345)

PR, IP: PR

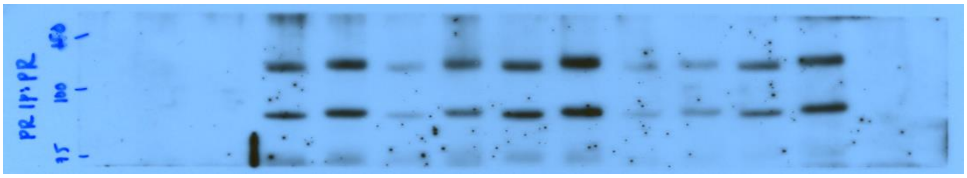

PR-B

PR-A

Supplementary Figure 3

PR

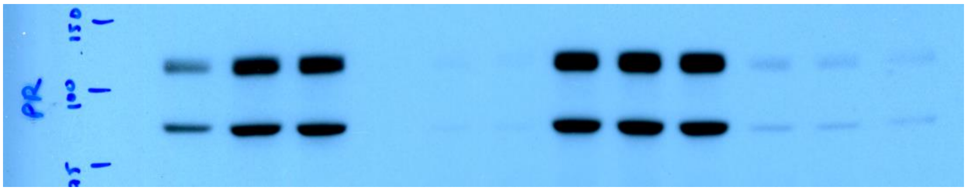

PR-B

PR-A

ER

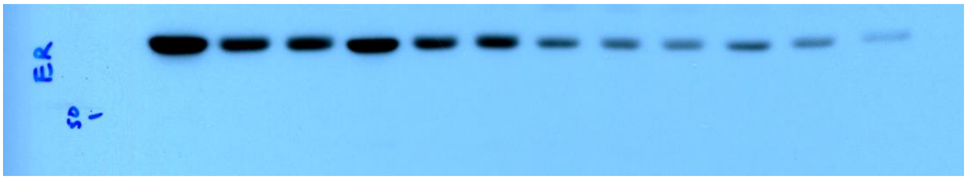

ER

GAPDH

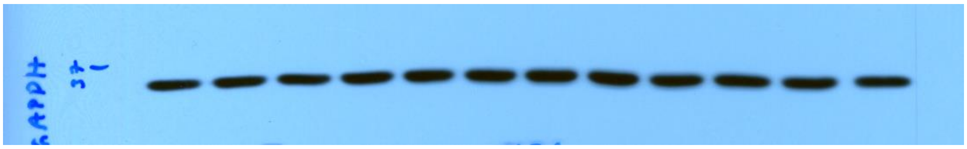

GAPDH

Supplementary Figure 6B

Phospho PR (Ser294)

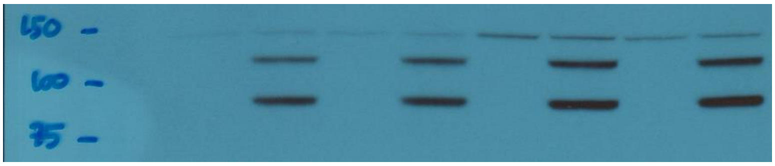

Non-Specific
Phospho PR-B (Ser294)
Phospho PR-A (Ser294)

PR

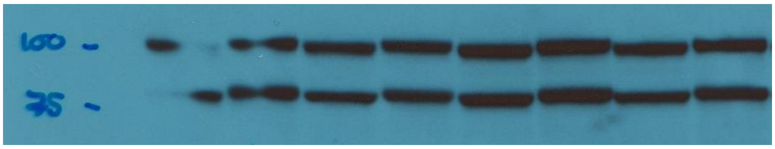

PR-B
PR-A

ER

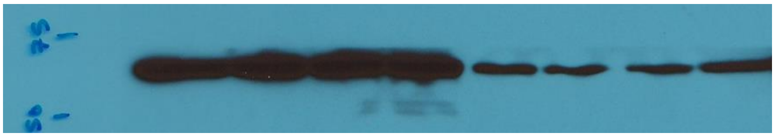

ER

GAPDH

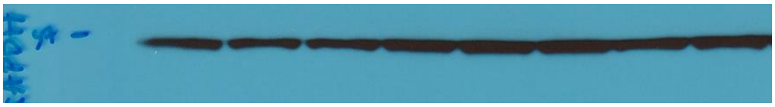

GAPDH

Supplementary Figure 6C

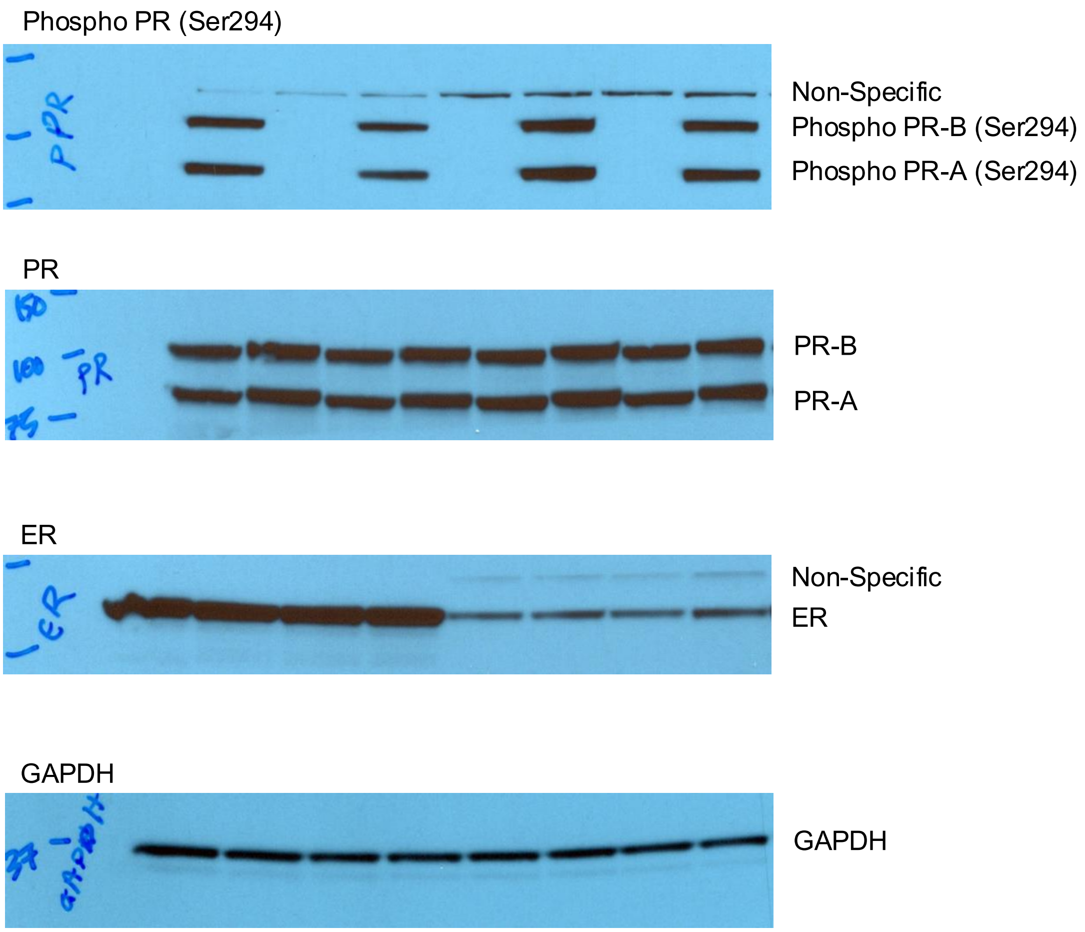

Supplementary Figure 6D

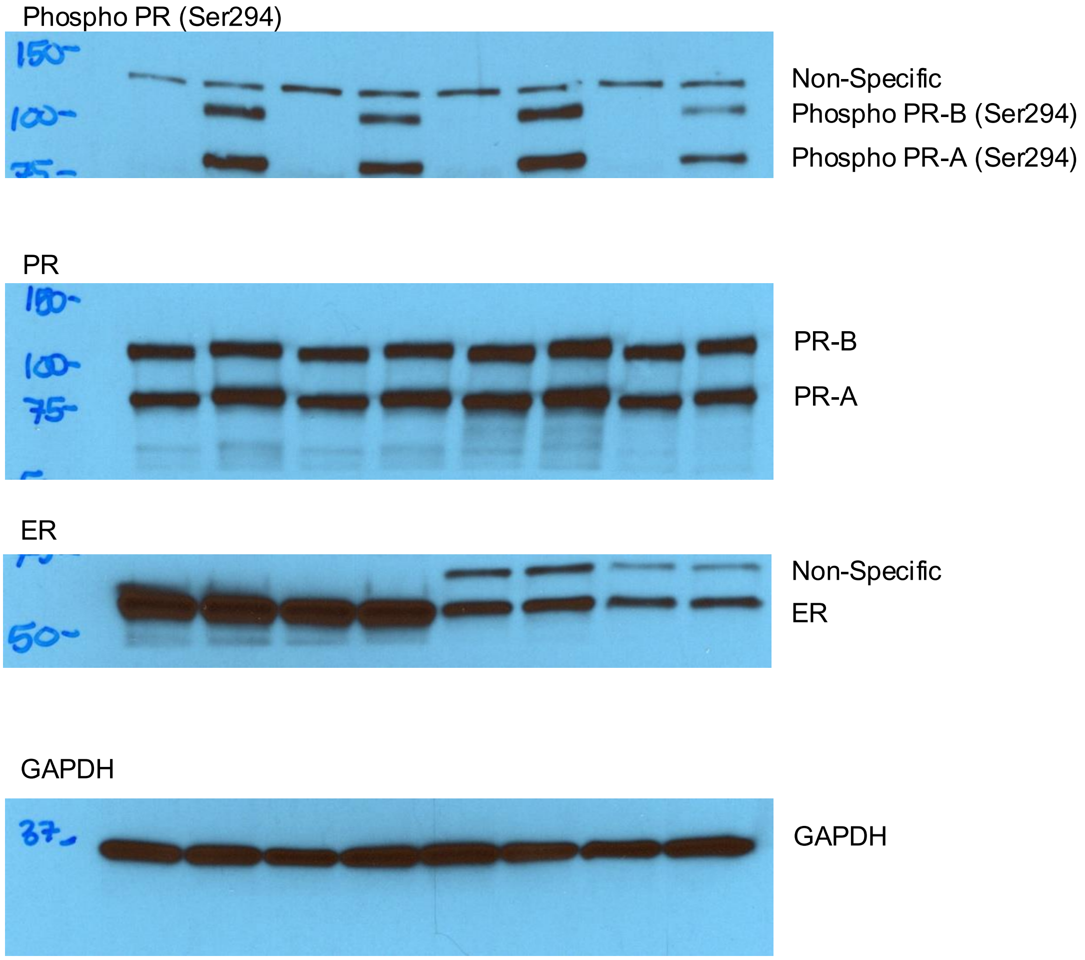

Supplement: Supplementary file 1 — Supplementary Material [file 41523_2026_939_MOESM1_ESM.pdf]
